# Molecular and Brain Volume Changes Following Aerobic Exercise, Cognitive and Combined Training in Physically Inactive Healthy Late-Middle-Aged Adults: The Projecte Moviment Randomized Controlled Trial

**DOI:** 10.3389/fnhum.2022.854175

**Published:** 2022-04-20

**Authors:** Alba Castells-Sánchez, Francesca Roig-Coll, Rosalía Dacosta-Aguayo, Noemí Lamonja-Vicente, Pere Torán-Monserrat, Guillem Pera, Alberto García-Molina, José Maria Tormos, Pilar Montero-Alía, Antonio Heras-Tébar, Juan José Soriano-Raya, Cynthia Cáceres, Sira Domènech, Marc Via, Kirk I. Erickson, Maria Mataró

**Affiliations:** ^1^Department of Clinical Psychology and Psychobiology, University of Barcelona, Barcelona, Spain; ^2^Institut de Neurociències, University of Barcelona, Barcelona, Spain; ^3^Unitat de Suport a la Recerca Metropolitana Nord, Fundació Institut Universitari per a la Recerca a l’Atenció Primària de Salut Jordi Gol i Gurina, Mataró, Spain; ^4^Institut d’Investigació en Ciències de la Salut Germans Trias i Pujol (IGTP), Badalona, Spain; ^5^Institut de Recerca Sant Joan de Déu, Esplugues de Llobregat, Spain; ^6^Department of Medicine, Universitat de Girona, Girona, Spain; ^7^Institut Guttmann, Institut Universitari de Neurorehabilitació, Universitat Autònoma de Barcelona, Badalona, Spain; ^8^Department of Neurosciences, Hospital Universitari Germans Trias i Pujol, Badalona, Spain; ^9^Institut de Diagnòstic per la Imatge, Hospital Germans Trias i Pujol, Badalona, Spain; ^10^Department of Psychology, University of Pittsburgh, Pittsburgh, PA, United States; ^11^Discipline of Exercise Science, College of Science, Health, Engineering and Education, Murdoch University, Murdoch, WA, Australia

**Keywords:** aerobic exercise, computerized cognitive training, combined training, brain volume, BDNF, neuroinflammation

## Abstract

Behavioral interventions have shown promising neuroprotective effects, but the cascade of molecular, brain and behavioral changes involved in these benefits remains poorly understood. Projecte Moviment is a 12-week (5 days per week—45 min per day) multi-domain, single-blind, proof-of-concept randomized controlled trial examining the cognitive effect and underlying mechanisms of an aerobic exercise (AE), computerized cognitive training (CCT) and a combined (COMB) groups compared to a waitlist control group. Adherence was > 80% for 82/109 participants recruited (62% female; age = 58.38 ± 5.47). In this study we report intervention-related changes in plasma biomarkers (BDNF, TNF-α, HGF, ICAM-1, SDF1-α) and structural-MRI (brain volume) and how they related to changes in physical activity and individual variables (age and sex) and their potential role as mediators in the cognitive changes. Our results show that although there were no significant changes in molecular biomarker concentrations in any intervention group, changes in ICAM-1 and SDF1-α were negatively associated with changes in physical activity outcomes in AE and COMB groups. Brain volume changes were found in the CCT showing a significant increase in precuneus volume. Sex moderated the brain volume change in the AE and COMB groups, suggesting that men may benefit more than women. Changes in molecular biomarkers and brain volumes did not significantly mediate the cognitive-related benefits found previously for any group. This study shows crucial initial molecular and brain volume changes related to lifestyle interventions at early stages and highlights the value of examining activity parameters, individual difference characteristics and using a multi-level analysis approach to address these questions.

## Introduction

*Being active might be your best health ally.* Current scientific literature states that an active lifestyle might have a positive impact on most hallmarks of aging, especially for brain and cognitive health (Ji et al., 2021). Behavioral interventions such as aerobic exercise (AE) and cognitive training programs are promising approaches to maintain and enhance cognition (Phillips, 2017; Sprague et al., 2019). Systematic reviews generally agree on the small-to-moderate effect size and consistent effects of AE on executive function, attention and speed (Colcombe and Kramer, 2003; Barha et al., 2017; Northey et al., 2018; Stillman et al., 2020) and the benefits of cognitive training in the trained domains but limited transfer to non-trained domains (Sprague et al., 2019). Combining both interventions showed promising results about the potential for greater effects on cognition (ten Brinke et al., 2020) yet recent systematic reviews reported that the extent of the combined effects were not significantly different from the individual programs (Gavelin et al., 2020; Guo et al., 2020). Studies including these approaches are examining biological mechanisms that could inform associations in prior studies. Identifying the molecular/cellular and brain changes related to exercise and cognitive training benefits, applied individually or in combination, is key to better understanding and prescribing these interventions as well as elucidating which combination of strategies would be more successful.

AE-related cognitive benefits are the result of multiple biological and psychosocial changes which were organized in a three-level model by Stillman et al. (2016). Molecular and cellular changes are described at Level 1 and include key factors involved in neuroplasticity, angiogenesis, metabolism and inflammation. For example, brain derived neurotrophic factor (BDNF) seems sensitive to AE (Knaepen et al., 2010). While available evidence consistently reported increased BDNF levels after an acute bout of exercise, the effect of regular exercise on resting BDNF remains unclear (Walsh and Tschakovsky, 2018). Initial systematic reviews and meta-analyses suggested that exercise training led to elevated peripheral BDNF (Knaepen et al., 2010; Coelho et al., 2013; Szuhany et al., 2015; Dinoff et al., 2016), but these results were driven by trials including clinical populations (Walsh and Tschakovsky, 2018). Recent reviews including healthy samples have reported non-significant effects of low to moderate AE on BDNF levels (Marinus et al., 2019) and conclude that evidence in human studies is still insufficient for making strong conclusions (Loprinzi, 2019). Other growth factors such as insulin growth factor (IGF) and vascular endothelial growth factor (VEGF) have consistently been related to endothelial cell proliferation and vessel growth as well as enhanced neurogenesis in the hippocampus after exercise due to their interaction with BDNF (Cotman et al., 2007; Stimpson et al., 2018). Molecular markers modulated by IGF and VEGF, such as stromal cell derived factor 1 (SDF1) or intercellular adhesion molecule 1 (ICAM-1) were suggested to be involved in the promotion of angiogenesis in the brain after exercise based on animal studies (Stimpson et al., 2018). Significantly decreased levels of ICAM-1 related to physical activity were found in patients with cardiovascular risk factors (Palmefors et al., 2014) but evidence in healthy samples is still inconclusive with some (Abd El-Kader et al., 2019) showing significant and others (Mohammadi et al., 2018) non-significant changes in post-test vs. pre-test comparisons. In healthy young men, SDF1 levels increased in response to acute exercise with an increase of endothelial cells in a 24 h follow-up (Chang et al., 2015). However, the effect of exercise programs on these markers in humans is still insufficient for drawing clear conclusions (Palmefors et al., 2014). Another growth factor potentially involved in this cascade of changes is the hepatocyte growth factor (HGF) which is a hepatokine primarily linked to liver regeneration. HGF has an anti-inflammatory role in adipose tissue and is linked to insulin resistance and diabetes (Oliveira et al., 2018). However, the effect of exercise on HGF levels has been scarcely addressed. Yasuda et al. (2004) found increased HGF levels after acute exercise which were negatively related to cardiovascular fitness (CRF) in patients after acute myocardial infarction. Exercise is known to also induce antioxidant and anti-inflammatory actions by modulating cytokines and oxidative stress factors on adipose tissue, body muscles and immune system (Sallam and Laher, 2016). Recent meta-analyses reported significant benefits on the pro-inflammatory markers in healthy populations after AE interventions (Monteiro-Junior et al., 2018; Zheng et al., 2019). However, evidence is still unclear about how exercise might influence each one of these markers. For example, Monteiro-Junior et al. (2018) and Zheng et al. (2019) agree on reduced levels of C-reactive protein and interleukin 6, but only Zheng et al. (2019) found significant effects for decreased TNF-α.

Changes at the molecular level are involved in the structural and functional brain benefits related to exercise as described at Level 2 by Stillman et al. (2016). In relation to brain volume, systematic reviews reported that AE training could be effective to prevent brain volume loss, increase gray matter volume (Erickson et al., 2014) and have a positive but small impact on global white matter volume (Sexton et al., 2016). Most of the research on this topic has focused on the impact of exercise on the hippocampus showing significant positive effects specifically on left hippocampal volume (Firth et al., 2018), specifically in older populations (>65 years) and in interventions that lasted over 24 weeks (Wilckens et al., 2020). However, there are still inconsistencies among trials including healthy samples. For example, increased local gray matter volumes in prefrontal (Colcombe et al., 2006; Ruscheweyh et al., 2011), cingulate (Ruscheweyh et al., 2011) and temporal cortices (Colcombe et al., 2006) and hippocampus (Erickson et al., 2011; Rosano et al., 2017) have been observed after long-term AE interventions, whereas non-significant changes in these same areas have been observed in trials lasting only 12 weeks (Maass et al., 2015; Matura et al., 2017; Sexton et al., 2020). This fact is in accordance with evidence highlighting how FITT-VP (Frequency, Intensity, Time, Type, Volume and Progression) parameters of exercise programs might be modulating the effect of the intervention on biomarkers at either at Level 1 or 2 (Cabral et al., 2019; Chen et al., 2020). Moreover, current literature suggests that individual characteristics of participants such as age, sex or genetics (Barha et al., 2019; Stillman et al., 2020) interplay with the intervention modulating the cognitive benefits related to exercise.

The Hebb (1949) principle has been used to generate hypotheses about the mechanisms involved in cognitive benefits related to cognitive training. Many authors have suggested that the regular practice of a cognitive task would repeatedly and simultaneously activate a group of neurons and, in turn, strengthen synaptic connections (Taya et al., 2015; ten Brinke et al., 2017; Gates et al., 2020). Animal studies using an “enriched environment” revealed increased long-term potentiation and enhanced BDNF levels in the hippocampus which led many authors to hypothesize a similar pattern in humans (Valenzuela and Sachdev, 2009). While cognitive training induces a significant increase of BDNF in patients with risk of dementia (Damirchi et al., 2017), evidence is still unclear if this effect occurs in healthy populations. There is evidence showing significant increases of BDNF after cognitive training programs lasting 5 (Ledreux et al., 2019) and 7 weeks (Rahe et al., 2015) and, at the same time, other trials did not find significant changes in BDNF after 10 (Küster et al., 2017) and 12 weeks (Tarassova et al., 2020). To our knowledge, other molecular and cellular mechanisms related to cognitive training effects have been scarcely addressed in healthy or clinical human populations. However, based on the overlapping effects of BDNF, IGF, and VEGF in the promotion of neuronal growth in the brain (Cotman et al., 2007), it could be suggested that these growth factors and other related molecular factors such SDF1 might also mediate cognitive training effects. For example, given the negative effect of pro-inflammatory cytokines on IGF signaling, which may alter protein synthesis in the brain (Cotman et al., 2007), we might expect a complex interaction between multiple factors at this level. Szabó et al. (2014) found that the number of stressful events and nuclear factor-jB, which is related to pro-inflammatory cytokines, predicted the increase of the right hippocampus after a videogame-based cognitive training approach. Therefore, understanding cognitive training related changes occurring at the molecular and cellular level could help to interpret any observed benefits in the structure and function of the brain. Few studies have published structural brain changes associated with cognitive interventions in healthy older adults (Belleville and Bherer, 2012). A systematic review (ten Brinke et al., 2017) reported mixed results from only three trials reporting changes in brain volume in healthy older adults. Lampit et al. (2015) found an increase in grey matter density in the right post-central gyrus in the cognitive training group compared to an active control group. However, Heinzel et al. (2014) and Antonenko et al. (2016) found no significant changes in grey matter and hippocampal volume, respectively. As suggested above, the heterogeneity of the findings at a microscopic and macroscopic level could be related to parameters of the training program and sample characteristics (Taya et al., 2015; Gates et al., 2020). The cascade of molecular mechanisms of cognitive training-induced neuroplasticity in healthy older adults remains understudied and the research on structural brain changes is still limited (ten Brinke et al., 2017; Gates et al., 2020).

Current research has focused on whether the positive impact of exercise on growth factors, inflammatory profile and brain structure might facilitate the neuroprotective effect of cognitive training when they are combined (Joubert and Chainay, 2018). In relation with BDNF, Rahe et al. (2015) found increased peripheral BDNF from pre to post-testing when cognitive training was applied alone or in combination with physical training in a 7-week program. Anderson-Hanley et al. (2012) reported that 3 months of cybercycling that included a cognitive component induced greater changes in BDNF levels compared to traditional cycling. The effects of a combined training (COMB) program on markers of inflammation have been studied in mice showing decreased TNF-α in the hippocampus. Related to effects on brain structure, Lövdén et al. (2012) reported that healthy older men engaging in a spatial navigation task (navigation task + walking) for 4 months maintained stable hippocampal volumes after the intervention compared to a control group which showed volume decrements consistent with age-related decline. However, a 7-month program of combined cognitive and AE training in a sample with mild cognitive impairment (MCI) found no effect on grey matter loss (Train the Brain Consortium, 2017). Therefore, despite the hypothesis of the potential additive effects of both interventions on neurobiological measures, there is a great degree of mixed evidence and more research in humans is needed.

Projecte Moviment is a randomized controlled trial about the effect of a high-frequency (5 days per week) short-term (12 weeks) program of AE, computerized cognitive training (CCT) and their combination in healthy physically inactive older adults (Castells-Sánchez et al., 2019). The observed changes on cognition, psychological status and physical activity outcomes have been published in Roig-Coll et al. (2020). In this study, we aim to examine the effect of the interventions on BDNF levels, markers of inflammation and volume changes in relevant brain areas compared to healthy controls. Secondly, we aim to test whether significant changes in physical activity outcomes are related to changes in molecular markers and brain volume. Finally, we aim to assess the moderating role of sex and age on molecular and brain volume changes and the possibility that changes in molecular and brain volume outcomes mediate the relationship between the intervention and cognitive benefits.

Correspondingly, we hypothesize that: (1) Intervention of physical activity, alone or combined with computerized cognitive training, would result in lower levels of inflammatory markers, increased BDNF, and greater brain volume. Computerized cognitive training, alone or combined with physical activity, would result in increased BDNF levels, and greater brain volume. (2) Sex and age would have a significant moderating role in the effects of the intervention on Level 1 and 2 biomarkers. (3) Levels 1 and 2 biomarkers would mediate significant intervention- related changes on cognition.

## Materials and Methods

### Study Design

Projecte Moviment is a multi-center, single-blind, proof-of-concept RCT which took place between November 2015 and April 2018 (ClinicalTrials.gov; NCT03123900). Participants were assigned to four parallel groups: an AE group, a CCT group, a COMB group and a waitlist control group. Interventions lasted 12 weeks and there were assessments at baseline and trial completion. The study was developed by the University of Barcelona in collaboration with Institut Universitari d’Investigació en Atenció Primària Jordi Gol, Hospital Germans Trias i Pujol and Institut Guttmann, and approved by the responsible ethics committees (Bioethics Commission of the University of Barcelona –IRB00003099- and Clinical Research Ethics Committee of IDIAP Jordi Gol -P16/181-) following the Declaration of Helsinki.

This research paper follows the previously published protocol (Castells-Sánchez et al., 2019) and results on the primary hypothesis (Roig-Coll et al., 2020) in accordance with the recommendations of CONSORT Statement.

### Participants

We recruited healthy adults aged 50–70 years old from the Barcelona metropolitan area using multiple strategies (lists of patients of general physicians, volunteers from previous studies, oral presentations in community centers, advertisements and local media). We informed and screened those interested over the phone and in an on-site interview. Individuals meeting inclusion and exclusion criteria (see Table 1) signed a written informed consent prior to study involvement. We further assessed the participant with a comprehensive neuropsychological battery, and we discarded subjects suggestive of MCI (1.5 SD below the normative data in any domain) regardless of their generic eligibility.

**TABLE 1:** Inclusion and exclusion criteria for Projecte Moviment.

Inclusion criteria	Exclusion criteria
Aged 50–70 years	Current participation in any cognitive training activity or during last 6 months > 2 h/week
<120 min/week of physical activity during last 6 months	Diagnostic of dementia or mild cognitive impairment
Mini-Mental State Examination (MMSE) ≥ 24	Diagnostic of neurological disorder: stroke, epilepsy, multiple sclerosis, traumatic brain injury, brain tumor
Montreal Cognitive Assessment 5-min (MoCA 5-min) ≥ 6	Diagnostic of psychiatric illness current or during last 5 years
Competency in Catalan or Spanish	Geriatric Depression Scale (GDS-15) > 9
Adequate visual, auditory and fine motor skills	Consumption of psychopharmacological drugs current or during last 5 years; or more than 5 years throughout life
Acceptance of participation in the study and signature of the informed consent	History of drug abuse or alcoholism current or during last 5 years; or more than 5 years throughout life; > 28 men and > 18 woman unit of alcohol/week
	History of chemotherapy
	Contraindication to magnetic resonance imaging

*MMSE (Blesa et al., 2001); MoCA 5-min (Wong et al., 2015); GDS-15 (Martínez et al., 2002).*

Participants were randomized after the baseline assessments and assigned to AE, CCT, COMB, and control groups. The allocation sequence was designed by a statistician and consisted of a random combination of sex, age and years of education allowing for balanced groups accounting for these demographic variables. The intervention team was responsible for the allocation and assessors remained blind to group assignment.

### Interventions

Interventions were home-based and scheduled for 5 days per week for 12 weeks. Participants randomized to AE group followed a progressive brisk walking program (Week 1: 30 min per day at 9–10 on the Borg Rating of Perceived Exertion Scale (BRPES; Borg, 1982) perceived as light intensity. Week 2: 45 min per day at 9–10 on BRPES. Weeks 3–12 (10 weeks): 45 min per day at 12–14 on BRPES perceived as moderate-high effort). Participants randomized to CCT group performed multimodal cognitive training using the Guttmann Neuropersonal Trainer online platform (GNPT^®^, Spain; Solana et al., 2014, 2015) in sessions of 45 min targeting executive function, visual and verbal memory and sustained, divided and selective attention. The demand of the tasks for each participant was adjusted by GNPT platform based on baseline cognitive performance and the ongoing scores of the activities. Participants randomized to COMB group performed the brisk walking program and the CCT as described above, separately, in single continuous bouts of 45 min for each intervention 5 days per week without order or time-point restrictions. Participants randomized to the control group were on the wait list for 12 weeks and were asked not to alter their regular lifestyle.

Participants monitored their activity in a diary registering the date and duration of the activity and any adverse events occurring as well as the intensity of the walking in BRPES units. Participants had the contact information for the intervention team who followed the intervention (phone calls every 2 weeks and a mid-point visit and a final visit) and ensured participation, solved inconveniences and barriers and obtained adherence based on participants feedback and platform data.

The protocol for each intervention condition is explained in more detail elsewhere (Castells-Sánchez et al., 2019).

### Assessment

All assessments were conducted in clinical environments at baseline within 2 weeks prior to the start of the intervention and, again, within 2 weeks after the completion of the program (Castells-Sánchez et al., 2019).

#### Blood Sample: Biomarkers at Level 1

Nurses in the Primary Health Care Centers obtained blood samples from the antecubital vein in EDTA tubes for plasma analyses between 8:00 and 9:00 a.m. All participants were instructed to fast overnight and not exercise 8 h before the blood test. Tubes were immediately transferred to the IGTP-HUGTP Biobank integrated in the Spanish National Biobanks Network of Instituto de Salud Carlos II (PT13/0010/0009) and Tumor Bank Network of Catalonia. They were processed following standard operating procedures with the appropriate approval of the Ethical and Scientific Committees and plasma aliquots were stored at −80^°^C.

We obtained peripheral BDNF levels using an ELISA kit (Human Free BDNF Quantikine ELISA Kit; R&D Systems, Minnesota, United States). The Proteome Profiler Human™ XL Cytokine Array was used to semi-quantitatively analyze a panel of 105 targeted cytokines (R&D Systems, MN, United States). Based on the results of the array and previously cited literature, TNF-α, ICAM-1, HGF, SDF1-α levels were selected to be quantitatively analyzed using the corresponding ELISA immunoassay method (Human TNF-α Quantikine HS ELISA, Human ICAM-1/CD54 Allele-specific Quantikine ELISA Kit, Human HGF Quantikine ELISA Kit, Human CXCL12/SDF-1 alpha Quantikine ELISA Kit; R&D Systems, Minnesota, United States).

#### Neuroimaging: Biomarkers at Level 2

Structural MRI data was collected at the Hospital Germans Trias i Pujol using a 3T Siemens Magnetom Verio Symo MR B17 (Siemens 243 Healthineers, Erlangen, Germany). We acquired T1-weighted multi-planar reformat sequences (acquisition time: 5:26 min, voxel: 0.9 × 0.9 × 0.9 mm, TR/TE/TI: 1900/2.73/900 ms, flip angle: 9^°^, slices: 192; thickness: 0.9 mm) and visually checked by an expert neuroradiologist. We used MRICloud^1^ (Mori et al., 2016) to analyze brain images performing a fully automated parcellation of 287 volumes based on multiple atlases that fuses different algorithms (Oishi et al., 2009) with a local search algorithm (Coupé et al., 2011). For our sample, we used atlas library version 10A, which includes 30 atlases from cognitively-normal individuals and individuals with cognitive impairment or dementia. We summed volumes of the brain tissue (Left Hemisphere + Right Hemisphere + Brainstem + Cerebellum) and CSF (Ventricles + Sulci) for calculated intracranial volume (ICV). Based on previous literature (Erickson et al., 2014; Stillman et al., 2016) and consistent with our recently published results (Roig-Coll et al., 2020), we obtained partial brain volumes of the following relevant areas: ventricles, total WM and GM of the frontal lobe, dorsolateral prefrontal cortex, cingulate cortex, parietal lobe, precuneus, temporal lobe and hippocampus.

#### Physical Activity

Physical activity levels were evaluated with the Minnesota Leisure Time Physical Activity Questionnaire (VREM; Ruiz et al., 2012) which asks about frequency and duration of multiple activities -sportive walking, sport/dancing, gardening, climbing stairs, shopping walking and cleaning house- during the last month. We calculated the energy expenditure for each activity transforming hours per month into units of metabolic equivalent of tasks (METs). We added the METs spent in sportive walking and sport/dancing activities to obtain a measure of Sportive Physical Activity (S-PA) and the METs spent in gardening, climbing stairs, shopping walking and cleaning house to obtain a measure of Non-Sportive Physical Activity (NS-PA).

#### Cardiorespiratory Fitness

CRF was assessed using the Rockport 1-Mile Test in which participants were instructed to walk one mile on a treadmill adjusting their speed in order to be as fast as possible without running. We used the standard equation reported by Kline et al. (1987) to estimate the maximal aerobic capacity (VO_2_max). The equation uses the following variables to estimate VO_2m*ax*_: weight, age, sex, time to complete the mile, and heart rate at the end of the test.

#### Cognitive Performance

An extensive cognitive assessment was conducted before the CRF test or any type of exercise in order to control for the effect of acute exercise on cognitive performance. The neuropsychological battery was constructed using a theoretically driven (Strauss and Spreen, 1998; Lezak et al., 2012) selection of tests addressing multiple cognitive functions: Flexibility (Trail Making Test B-A time; Tombaugh, 2004), Fluency (letter and category fluency; Peña-Casanova et al., 2009), Inhibition (interference-Stroop Test; Golden, 2001), Working Memory (backward-WAIS-III; Wechsler, 2001), Visuospatial Function (copy accuracy-Rey Osterrieth Complex Figure; Rey, 2009), Language (Boston Naming Test-15; Goodglass et al., 2001), Attention (forward span, digit symbol coding and symbol search WAIS-III; Wechsler, 2001), Speed (Trail Making Test-A; Tombaugh, 2004; copy time-Rey Osterrieth Complex Figure; Rey, 2009), Visual Memory (memory accuracy-Rey Osterrieth Complex Figure; Rey, 2009) and Verbal Memory (total learning and recall-II Rey Auditory Verbal Learning Test; Schmidt, 1996). Six general domains were designed: (1) Executive Function, (2) Visuospatial Function, (3) Language, (4) Attention-Speed, (5) Memory, and (6) Global Cognitive Function. Extended details in Supplementary Table 1.

### Statistical Analysis

We conducted statistical analyses using Statistical IBM SPSS Statistics 24. First, we ensured data quality by examining the distribution of raw scores (i.e., outliers, skewness). We calculated the normative mean and standard deviation for each variable based on the full sample and used them to convert raw data to z-score (in comparison with the normative data) for each participant. Then, we calculated change scores (post-test minus pre-test), compared baseline scores between groups and performed cross-time partial correlations to detect potential confounds and ceiling effects.

Change between baseline and follow-up within group was examined using a *t*-test of related samples. In order to compare each intervention group to the control group we regressed change in each outcome of interest on the baseline outcome score, sex, age, years of education, BMI, and the *dummy treatment variables.* ICV was included as a covariate for the models including brain volume outcomes.

We addressed whether the significant intervention-related changes in S-PA and CRF observed in the AE and COMB groups previously published in Roig-Coll et al. (2020) were related to change in biomarkers at Levels 1 and 2 applying partial correlations adjusted by sex, age, years of education, BMI and, for brain volumes, ICV was added.

We used the PROCESS macro for SPSS (Hayes, 2017) to analyze the moderating effect of age and sex on intervention-related changes for biomarkers at Levels 1 and 2. We also used the PROCESS macro to perform mediation analyses to assess whether change in biomarkers at Level 1 or 2 mediated the cognitive benefits observed in the AE and COMB groups (Roig-Coll et al., 2020). For mediation analyses the independent variable was a treatment variable (condition vs. control), the dependent variables were change in cognition for those functions that showed significant intervention-related changes. We used as mediators the change in biomarkers at Levels 1 and 2 controlling for baseline performance score, age, sex, years of education, BMI and, when biomarkers at Level 2 were introduced as mediators, ICV was added. These analyses were computed with bias-corrected bootstrapped 95% confidence intervals (CIs) based on 5,000 bootstrap samples. Significance of mediation was indicated if the CIs in Path AB did not overlap with 0 (Hayes, 2017).

## Results

### Participants

**FIGURE 1:**
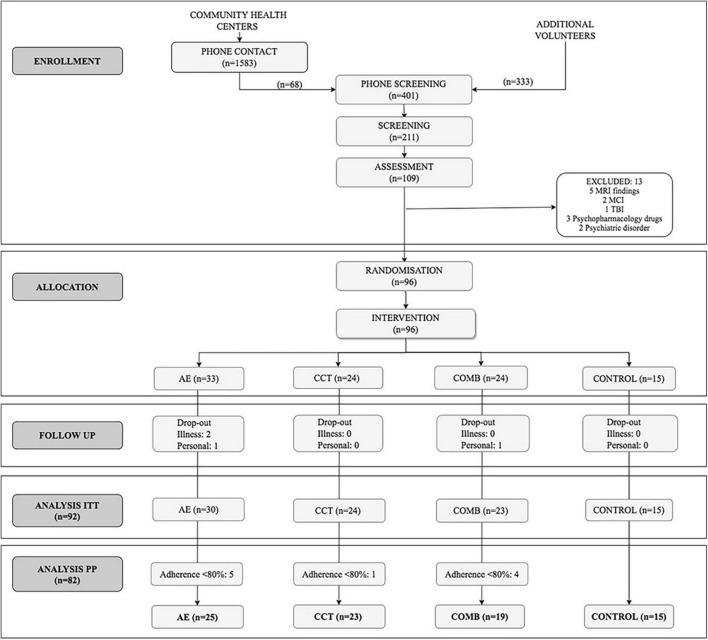
CONSORT flow diagram.

We conducted analyses in the Per Protocol (PP) sample which included 82 subjects with a level of adherence > 80% (*n* = 82, 62% female; age = 58.38 ± 5.47; see Table 2) out of the 109 who completed the baseline assessment and the 92 who completed the intervention (intention to treat, ITT) (see Figure 1; extended details on Roig-Coll et al., 2020). There were no significant differences in demographic variables between groups in the ITT sample and between the ITT and PP sample (see Supplementary Tables 2, 3).

**TABLE 2:** Participants characteristics in the PP sample at baseline.

	Total Mean (SD)	AE Mean (SD)	CCT Mean (SD)	COMB Mean (SD)	Control Mean (SD)	Comparison Group
n total/n females	82/51	25/13	23/16	19/14	15/8	*X^2^*(3) = 3.20, *p* = 0.361
Age (years)	58.38 (5.47)	58.40 (5.12)	57.91 (5.31)	60.32 (5.54)	56.60 (5.97)	H(3) = 3.53, *p* = 0.317
Years of education (years)	12.52 (5.57)	12.44 (5.75)	12.04 (4.94)	12.37 (5.43)	13.60 (6.72)	H(3) = 0.28, *p* = 0.963
Vocabulary subtest (direct score-WAIS-III)	44.14 (8.30)	43.92 (9.53)	44.26 (7.16)	44.53 (8.02)	43.80 (8.98)	F(3,77) = 0.03, *p* = 0.993
MMSE	28.21 (1.31)	28.20 (1.19)	28.09 (1.51)	28.47 (1.26)	28.07 (1.34)	H(3) = 1.23, *p* = 0.747
BMI (kg/m^2^)	28.63 (4.96)	28.14 (5.53)	28.13 (4.26)	28.37 (4.42)	30.49 (5.70)	H(3) = 1.72, *p = 0.*632

*AE, Aerobic Exercise group; CCT, Computerized Cognitive Training group; COMB, Combined group; MMSE, Mini-Mental State Examination; WAIS-III, Wechsler Adult Intelligence Scale. Mean (SD); X^2^, chi square; H, Kruskall Wallis H test; F, Anova test. See Supplementary Table 4 for molecular and brain volume Baseline outcomes.*

The PP sample showed no significant baseline differences between groups in demographic, molecular, brain volume, physical and cognitive outcomes except for SDF1-α and NS-PA (extended details in Supplementary Table 4). SDF1-α at baseline was included as a covariate (extended details in Supplementary Table 4).

### Intervention-Related Changes in Molecular Biomarkers

There were no significant changes in molecular biomarkers between baseline and follow-up in any group. Contrasts between each intervention and control group for molecular biomarker outcomes are reported in Table 3. The results for these outcomes showed no significant changes in AE, CCT and COMB groups compared to control for any molecular biomarker outcome.

**TABLE 3:** Intervention related changes in molecular biomarkers (Level 1).

Molecular biomarkers	AE vs Controls B (95%CI), SMD, *p-*Value	CCT vs Control B (95%CI), SMD, *p-*Value	COMB vs Control B (95%CI), SMD, *p-*Value
BDNF (pg/ml)	−1293.54, (−4333.82, 1746.74), SMD = −0.13, *p* = .398	−1081.93 (−3925.15, 1761.30), SMD = −0.11, *p* = .450	−128.39 (−3235.57, 2978.79), SMD = −0.01, *p* = .934
TNF-α (pg/ml)	0.08 (−0.06, 0.21), SMD = 0.16, *p* = .287	<0.01 (−0.14, 0.14), SMD <0.01,*p* = .990	−0.03 (−0.18, 0.12), SMD = −0.07, *p* = .663
HGF (pg/ml)	24.81 (−102.48, 152.10), SMD = 0.06, *p* = .669	2.47 (−123.84, 128.78), SMD = 0.01, *p* = .969	30.48 (−105.27, 166.23), SMD = 0.07, *p* = .656
ICAM-1 (ng/ml)	−4.54 (−22.39, 13.31), SMD = −0.08, *p* = .614	−0.50 (−18.19, 17.20) SMD = −0.01, *p* = .955	−9.04 (−27.94, 9.86), SMD = −0.14, *p* = .343
SDF1-a (pg/ml)	−38.11 (−211.95, 135.73), SMD = −0.06,*p* = .663	−48.23 (−221.07, 124.61), SMD = −0.08, *p* = .580	140.87 (−47.06, 328.79), SMD = 0.22, *p* = .139

*AE, Aerobic Exercise group; CCT, Computerized Cognitive Training; COMB, Combined Group; SMD, β. Covariates: sex, age, years of education, body mass index and baseline*

### Intervention-Related Changes in Brain Volume

There was a significant change in precuneus volume between baseline and follow-up for the CCT group (*t* = −2.62, *p* = 0.019). There were no significant changes between baseline and follow-up for the AE, COMB and control groups. Contrasts between each intervention and control group for brain volume outcomes are reported in Table 4. The results for these outcomes showed a significant change in frontal lobe and in precuneus volume in the CCT compared to control group. There were no significant changes in AE and COMB groups compared to control for any outcome.

**TABLE 4:** Intervention related changes in brain volumes (Level 2).

Brain volumes (mm^3^)	AE vs. Controls B (95%CI), SMD, *p*-value	CCT vs. Control B (95%CI), SMD, *p-*value	COMB vs. Control B (95%CI), SMD, *p*-value
Ventricles	−335.42, (−2176.65, 1505.81), SMD = −0.07, *p* = 0.716	−89.42 (−2020.52, 1841.69), SMD = −0.02, *p* = 0.926	348.67 (−1581.37, 2278.71), SMD = 0.06, *p* = 0.719
Total white matter	−598.23 (−2268.85, 1072.39), SMD = −0.12, *p* = 0.476	140.20 (−1654.67, 1935.08), SMD = 0.03, *p* = 0.876	−432.88 (−2216.83,1351.08), SMD = −0.08, *p* = 0.629
Frontal lobe	2462.00 (−1611.56, 6535.56), SMD = 0.21, *p* = 0.231	5031.79 (717.77, 9345.82), SMD = 0.41, *p* = 0.023*	3566.03 (−759.51, 7891.57), SMD = 0.29, *p* = 0.104
Dorsolateral prefrontal cortex	119.31 (−757.30, 995.92), SMD = 0.05, *p* = 0.786	567.92 (−352.66, 1488.50), SMD = 0.23, *p* = 0.221	220.64 (−691.70, 1132.98), SMD = 0.09, *p* = 0.630
Cingulate cortex	240.33 (−339.98, 820.64), SMD = 0.15, *p* = 0.410	387.42 (−227.20, 1002.05), SMD = 0.23, *p* = 0.212	281.35 (−332.97, 895.66), SMD = 0.16, *p* = 0.363
Parietal lobe	95.67 (−1843.45, 2034.79), SMD = 0.02, *p* = 0.922	1312.57 (−770.68, 3395.82), SMD = 0.23, *p* = 0.212	148.65 (−1888.81, 2186.12), SMD = 0.03, *p* = 0.884
Precuneus	34.64 (−453.43, 522.71), SMD = 0.02, *p* = 0.887	530.47 (21.65, 1039.28), SMD = 0.35, *p* = 0.041*	289.19 (−222.30, 800.68), SMD = 0.19, *p* = 0.262
Temporal lobe	55.82 (−2084.45, 2196.08), SMD = 0.01, *p* = 0.958	145.31 (−2100.17, 2390.79), SMD = 0.02, *p* = 0.897	−1385.20 (−3645.44, 875.04), SMD = −0.22, *p* = 0.225
Hippocampus	23.91 (−75.07, 122.89), SMD = 0.08, *p* = 0.630	15.56 (−88.00, 119.13), SMD = 0.05, *p* = 0.764	44.47 (−60.54, 149.47), SMD = 0.14, *p* = 0.400

AE, Aerobic Exercise group; CCT, Computerized Cognitive Training; COMB, Combined Group; SMD, β. Covariates: sex, age, years of education, body mass index, baseline and intracranial brain volume. *p < 0.05.

### Relation Between Biomarkers at Levels 1 and 2 and Changes in Physical Activity Outcomes

In our previous study, AE and COMB groups showed significant intervention-related changes in S-PA and CRF levels (Roig-Coll et al., 2020). In our current study, the increase of S-PA in the AE group was negatively associated with change in ICAM-1 levels (*r* = −0.55, *p* = 0.016). Results were not significant for changes in BDNF, TNF-α, HGF or SDF1-α. Moreover, the increased levels of CRF in the AE group was negatively associated with change in SDF1-α levels (*r* = −0.50, *p* = 0.057) and not significantly associated with the other biomarkers. In the COMB group, the significant change in S-PA was not related to changes in any of the measured molecular markers. However, the significant benefits on CRF were negatively related to change in ICAM-1 (*r* = −0.66, *p* = 0.020) and SDF1-α (*r* = −0.89, *p* = < 0.001) levels. Intervention-related changes in physical activity in AE and COMB groups were not significantly related to changes in any measure of brain volume.

### Sex and Age Moderation Effects on Biomarkers at Levels 1 and 2

**FIGURE 2:**
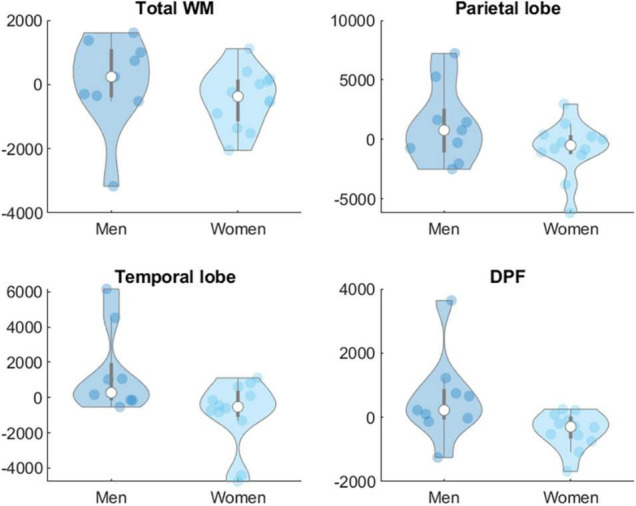
Sex difference in changes in brain volumes (Level 2) in mm^3^ for group AE. WM, white matter; DPF, dorsolateral prefrontal cortex.

**FIGURE 3:**
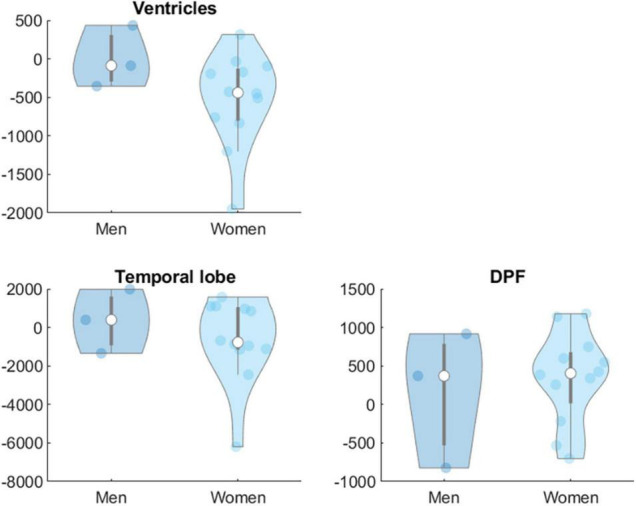
Sex difference in changes in brain volumes (Level 2) in mm^3^ for group COMB. DPF, dorsolateral prefrontal cortex.

Moderation analyses showed that age did not significantly moderate the effect of the intervention on biomarkers at Levels 1 and 2 in any group. Sex was not a significant moderator of the effects of the intervention on biomarkers at Level 1 in any group but showed significant effects on some of the targeted brain volume areas. In the AE group sex (women = 1, men = 0) moderated the effects of the intervention on total WM [β = −3133.51, *t*(53) = −2.45, *p* = 0.018], parietal [β = −3060.29, *t*(53) = −2.07, *p* = 0.043] and temporal lobe [β = −4028.46, *t*(53) = −2.47, *p* = 0.017] and dorsolateral prefrontal cortex volumes [β = −1819.39, *t*(53) = −2.89, *p* = 0.006] (Figure 2). In the COMB group sex also moderated the effects of intervention on ventricles [β = −5380.97, *t*(53) = −3.60, *p* = < 0.001], temporal lobe [β = 4690.43, *t*(53) = 2.56, *p* = 0.013] and dorsolateral prefrontal cortex [β = 1670.17, *t*(53) = 2.26, *p* = 0.028] (Figure 3).

### Mediation Effects on Intervention-Related Cognitive Benefits

Our previous study found significant changes in Executive Function, Attention, and Speed in one or more intervention groups (Roig-Coll et al., 2020). In our current study, we applied mediation analyses to observe whether changes in Levels 1 and 2 biomarkers mediated the association between the intervention and changes in these cognitive domains. Mediation analyses showed no significant indirect effect of BDNF, TNF-α, HGF, SDF1-α, and ICAM-1 levels and the targeted brain volume areas on the observed cognitive benefits for any group (BCa CI included zero for all analyses).

## Discussion

In this paper we report molecular and brain volume changes in the Projecte Moviment trial, which studied the effects of AE, CCT and their combination in healthy inactive late-middle-aged adults compared to a waitlist control group. Cognitive changes in Executive Function and Attention-Speed in the AE group and in Attention-Speed in the COMB group were previously published (Roig-Coll et al., 2020) and led us to address other micro and macroscopic correlates that might be involved in these interventions. We previously found that regular exercise was related to the inflammatory profile, brain volume and cognition in a cross-sectional sample (Castells-Sánchez et al., 2020, 2021).

In the present trial, baseline-to-follow-up changes in Level 1 biomarkers, including BDNF, were not significant within either group or in comparison with those in the control group, only changes in Level 1 biomarkers, such as ICAM-1 and SDF1-α, correlated with physical activity outcomes in groups including physical exercise (AE and COMB) indicating their relevance. At Level 2, brain volume biomarkers, the CCT group showed a significant intervention-related increase in the volume of the precuneus.

Participants in the AE group did not show significant within-group pre to post-test changes nor significant changes in comparison with the control group for any of the molecular correlates or targeted brain volume areas after 12 weeks in a 5 days per week, 45 min a day brisk walking program. The intervention-related results are in accordance with recent evidence stating that short-term trials typically do not report significant changes in neurotrophic factors or structural brain changes although they do in brain connectivity markers (Cabral et al., 2019; Wilckens et al., 2020). These results suggest that despite an increased frequency of activity during a 12-week program, the duration of the program might be more relevant for these molecular and structural brain changes since training programs lasting on the order of 6–12 months reported structural brain benefits (Colcombe et al., 2006; Erickson et al., 2011; Ruscheweyh et al., 2011). Therefore, our results suggest that the parameters of the activity, such as duration and intensity, are critical aspects of an exercise intervention. Another issue that might explain our results is that the sample was healthy and aged 50–70 years. As suggested by Erickson et al. (2014), the positive effects of exercise on gray matter volume might be significant in older age ranges in which there are greater brain volume losses. This suggestion could be extended to samples with pathology in which evidence tends to be more consistently significant (Wilckens et al., 2020).

Participants in the CCT group did not show significant changes on BDNF and, as expected, for any of the other targeted biomarkers at Level 1. The magnitude of the changes in BDNF might not be detectable yet over this duration and might require more prolonged exposure to cognitive training to induce a significant change. In addition, the parameters of the CCT program, the lack of pathology and age of our participants might also be playing a role as described for the AE interventions. Regarding markers at Level 2, the CCT group showed a significant intervention-related increase in the volume of the precuneus and compared to the change observed in the control group. These findings suggest that engaging in a multimodal CCT for 45 min a day, 5 days a week for 12 weeks might increase the volume of the precuneus which has a fundamental role in cognition: it is highly involved in the integration of tasks, visuo-spatial imagery, episodic memory retrieval as well as self-referential processing (Cavanna and Trimble, 2006). This result highlights current research challenges in this field since participants in the CCT group did not show significant changes in the cognitive assessments compared to the control group (Roig-Coll et al., 2020) in accordance with other trials of cognitive training (Boujut et al., 2020). The possible neuroprotective effects of a cognitive training program should be addressed by examining molecular, brain and cognitive markers given the challenge of detecting far transfer effects. We also found that change in the frontal lobe was also significant in the CCT group compared to the control group although neither participants in the CCT group nor the control group showed significant increase or loss in this area. In this case, we found significant results in the regression model but not when we performed the pre-to post t-test to see where the difference was and in which direction. These results indicate that there were changes with opposite directions in the two groups (i.e., CCT and control groups). Although these changes themselves were not significantly different from zero (i.e., non-significant pre-to-post change), they were significantly different across the two groups. The sufficiently large sample size provides considerable safeguard against selection bias. One can speculate, however, that our results indicate a slight, potentially age-related, change in the control group, against which the intervention provides protection.

Our study also shows that combining brisk walking and multimodal CCT for 12 weeks, 5 days per week in bouts of 45 min for each did not lead to greater benefits in the molecular biomarkers and brain volume areas analyzed pre to post-test and compared to the control group. As suggested for each intervention individually, parameters of the activity or characteristics of the sample might be responsible, though the scarcity of studies and high heterogeneity in the intervention characteristics (Anderson-Hanley et al., 2012; Lövdén et al., 2012; Rahe et al., 2015) make a challenge to draw firm conclusions. Moreover, determining whether a sequential or simultaneous strategy is best for cognitive benefits is still a controversy (Guo et al., 2020). Our findings might relate to the importance of the scheduled order of the interventions, specifically when interventions are applied simultaneously (Gheysen et al., 2018; Guo et al., 2020). Enhanced effects have been reported when exercising before the cognitive training suggesting that exercise facilitates an environment that enhances the effect of cognitive training.

We also found that significant changes in physical activity outcomes (amount of PA and CRF), found in the AE and COMB groups (Roig-Coll et al., 2020), were related to significant changes in immune, cardiovascular and brain health biomarkers. Increases in the amount of PA observed in the AE group and higher CRF in the COMB group were related to reduced levels of ICAM-1, which is a pro-inflammatory molecule promoted by IGF and TNF-α and highly expressed in endothelial cells and related to atherosclerosis (Che et al., 2002; Frank and Lisanti, 2008). Increase in CRF in the AE and COMB groups was also related to reduced levels of SDF1-α which is the most common variant of SDF, a chemokine protein involved in angiogenesis and brain tissue repair, that contributes to neuroinflammation which in higher concentrations can lead to toxic environments and clinical consequences (Guyon, 2014). These results suggest that a 12-week program of brisk walking, in combination with cognitive training or alone, might enhance markers associated with cardiovascular and inflammatory health.

Further analyses examined the potential moderating role of individual characteristics on the interventions given their important role when designing personalized programs. We did not find a significant moderation effect of age on intervention-related changes in biomarkers at Level 1 and Level 2 in any group. Despite evidence is scarce for CCT programs, previous papers reported interactions of age with the neuroprotective effect of exercise (Colcombe and Kramer, 2003; Erickson et al., 2014) suggesting that the effect could be more apparent at older ages due to greater brain volume losses. Moreover, the mediating role of CRF in exercise-related cognitive benefits has been found in participants aged 70 or older but not in younger (Bherer et al., 2021). Therefore, the lack of a significant moderating effect of age in our study could be related to the relatively younger age of our participants as well as the limited age range of our participants. In relation to sex, we did not detect any significant moderation effects of sex on intervention-related changes in molecular biomarkers (Level 1) in any group despite previous evidence suggesting that sex moderates the effect of exercise on BDNF levels (Szuhany et al., 2015). However, we found that sex moderated the effects of the intervention on brain volumes on AE (total WM, parietal, temporal, and dorsolateral prefrontal cortex) and COMB groups (ventricles, temporal and dorsolateral prefrontal cortex) suggesting that men may benefit more than women. These results are consistent with our previously published results (Castells-Sánchez et al., 2019, 2020, 2021) and the larger adaptations that men might experience after exercise, as suggested in previous research (Al-Mallah et al., 2016). Moreover, these findings add support to the literature highlighting biological sex as a relevant moderator of the relationship between exercise and brain health (Barha and Liu-Ambrose, 2018; Barha et al., 2019). Sex differences in the physical activity-induced cognitive and brain changes may be associated with specific physiological adaptations in women and men, such as changes in neuroplastic processes, hormones, neurotransmitter systems and neurotrophic factors (Barha et al., 2019). Projecte Moviment adds support to the recent and growing evidence suggesting that sex matters. However, larger samples are necessary to run more highly powered intra-group analyses and further research should address the mechanisms underlying cognitive and physical training effects stratified by sex.

Finally, we examined the potential mediation effect of changes in molecular biomarkers and brain volume of targeted areas in the intervention-cognitive benefits through testable models in order to add evidence about the mechanisms underlying the neuroprotective effect of behavioral interventions (Stillman et al., 2020). The results of these mediation analyses showed that changes in biomarkers at Levels 1 and 2 did not significantly mediate the observed cognitive benefits in AE (Executive Functions and Attention-Speed) and in COMB groups (Attention-Speed). Previous evidence including a 1-year AE intervention reported that change in BDNF levels mediated the effect of intervention-related cognitive (task-switch) benefits in older adults (>71 years) (Leckie et al., 2014) and that intervention-related increases in hippocampal volume were significantly correlated to improvements in memory performance (Erickson et al., 2011). Since our intervention programs lasted 12 weeks, the duration of the activity as well as other potential methodological issues might be key parameters to find consistent effects of these interventions on these molecular and structural brain outcomes and their effect on cognition. The length of the intervention could be an important factor not only due to the persistence of the activity but also for the time needed to observe molecular and structural changes in healthy subjects. We might also hypothesize that early intervention-related cognitive benefits might be mediated by changes in other molecular markers such as other growth factor, cytokines or hormones which might interact differently for women and men and in relation to brain outcomes such as increased white matter integrity and better functional connectivity (Cabral et al., 2019). The sample size is also an important issue for trials examining the mechanisms of interventions since large samples are needed to detect mediation effects when the outcome measure is highly variable (neuroimaging data, molecular biomarkers) (Stillman et al., 2016). Those questions should be addressed for COMB interventions including AE and CCT in order to better understand the role of each single intervention. New omics technology might shed light on the complexity of biological networks underlying the cognitive benefits related to behavioral interventions (Hoffman, 2017).

The cascade of changes initiated by behavioral interventions that lead to cognitive enhancements is complex and requires a combined multi-level approach that addresses moderators such as sex, age, pathology and genetics. Further research should overcome our limited intra-group sample and include a wider range of participants with age and sex balanced across groups in order to be able to make specific intra-group analyses. We also acknowledge potential desirability bias in our sample since we obtained self-reported adherence and participants were highly motivated, the interventions were individual and home-based, and the use of a waitlist control group.

### Limitations

Some limitations of the study should be addressed. First, the parameters of the activity such as duration or intensity might be relevant to detect changes in the targeted molecular and structural brain outcomes and we did not use actimeter, an objective measure of those variables, as we used self-reported measures of every participant instead. Further studies should consider the use of actimeter in this kind of studies. Second, other molecular and brain mechanisms should be addressed in order to better understand the cognitive benefits observed at early stages. Finally, our inferences on the neuroimaging findings have not been corrected for multiple comparison; therefore, they must be considered with caution. On the other hand, the overall direction of the results supports our findings and confirm their value.

## Conclusion

The significant relationship found between changes in physical activity outcomes and changes in immune, cardiovascular and brain health biomarkers highlight the role of the aerobic exercise in the promotion of a molecular cascade involving immunity and cardiovascular related markers that might enhance a neuroprotective effect in the aerobic exercise and combined groups. Our findings also highlight the importance of sex as a relevant variable for brain volume in these interventions. Our results provide further support for the effectiveness of CCT and reveals some underlying neurobiological mechanisms. CCT showed promising results enhancing the brain volume of the precuneus, despite previously published results that did not show significant changes in cognitive assessments. These results highlight the importance of a multi-level approach when assessing and describing the neuroprotective effect of behavioral interventions.

Moreover, parameters of the activity such as duration or intensity might be relevant to detect changes in the targeted molecular and structural brain outcomes. Other molecular and brain mechanisms should be addressed in order to better understand the cognitive benefits observed at early stages.

## Data Availability Statement

The raw data supporting the conclusions of this article will be made available by the authors, without undue reservation.

## Ethics Statement

The studies involving human participants were reviewed and approved by the Bioethics Commission of the University of Barcelona (IRB00003099) and Clinical Research Ethics Committee of IDIAP Jordi Gol (P16/181). The patients/participants provided their written informed consent to participate in this study.

## Author Contributions

MM conceptualized the study, contributed to the study design and implementation as Principal Investigator, and guided and supervised all the statistical analysis and writing of this manuscript. PT-M and KE made substantial contributions to the design and implementation of the trial. AC-S and FR-C contributed to the design and implementation of this trial, collaborated with recruitment and evaluated participants before and after interventions, analyzed the data, and wrote this manuscript. NL-V collaborated with recruitment and did the follow-up of the intervention groups. AG-M and JT assisted in the use of GNPT program for computerized cognitive training. GP guided and supervised the statistical analysis. RD-A made the revision of the present manuscript and answered to the reviewer s questions and contributed processing the neuroimaging data and MV with the management of molecular markers. PM-A, AH-T, JS-R, CC, and SD contributed to the implementation of the trial from their area of expertise. All authors reviewed the manuscript and provided final approval for publication of the content.

## Conflict of Interest

The authors declare that the research was conducted in the absence of any commercial or financial relationships that could be construed as a potential conflict of interest.

## Publisher’s Note

All claims expressed in this article are solely those of the authors and do not necessarily represent those of their affiliated organizations, or those of the publisher, the editors and the reviewers. Any product that may be evaluated in this article, or claim that may be made by its manufacturer, is not guaranteed or endorsed by the publisher.

## Footnotes

1
https://mricloud.org/
