# Supplementary material for: Molecular and Brain Volume Changes Following Aerobic Exercise, Cognitive and Combined Training in Physically Inactive Healthy Late-Middle-Aged Adults: The Projecte Moviment Randomized Controlled Trial
Source: Front Hum Neurosci. 2022 Apr 20;16:854175. doi: 10.3389/fnhum.2022.854175 (PMC9067321; doi:10.3389/fnhum.2022.854175)
Supplement: Supplementary file 1 [file Table_1.docx]

| **Table 1**  Cognitive outcomes: variables and measures | | | |
| --- | --- | --- | --- |
| Composites 1^st^ Level | Composites 2^nd^ Level | Tests - Subtest | Measure |
| Executive Function | Flexibility | TMT B -A | Z score |
|  | Fluency | Letter fluency | Z score |
|  |  | Category fluency | Z score |
|  | Inhibition | Stroop - Interference | Z score |
|  | Working Memory | WAIS III - Backward Span | Z score |
| Visuospatial Function | Visuospatial Function | ROCF - Copy Accuracy | Z score |
| Language | Language | BNT (15 items) | Z score |
| Attention - Speed | Attention | WAIS III - Forward Span | Z score |
|  |  | WAIS III - Digit Symbol Coding | Z score |
|  |  | WAIS-III - Symbol Search | Z score |
|  | Speed | TMT - A | Z score |
|  |  | ROCF - Copy Time | Z score |
| Memory | Visual Memory | ROCF - Memory Accuracy | Z score |
|  | Verbal Memory | RAVLT - Total Learning | Z score |
|  |  | RAVLT - Recall II | Z score |
| TMT, Trail Making Test (Tombaugh, 2004); Verbal Fluency Tests (Peña-Casanova et al., 2009); Stroop Test (Golden, 2001); WAIS-III, Wechsler Adult Intelligence Scale (Wechsler, 2001); ROCF, Rey-Osterrieth Complex Figure (Rey, 2009); BNT, Boston Naming Test (Goodglass et al., 2001); RAVLT, Rey Auditory Verbal Learning Test (Schmidt, 1996). | | | |
