# Supplementary material for: Molecular and Brain Volume Changes Following Aerobic Exercise, Cognitive and Combined Training in Physically Inactive Healthy Late-Middle-Aged Adults: The Projecte Moviment Randomized Controlled Trial
Source: Front Hum Neurosci. 2022 Apr 20;16:854175. doi: 10.3389/fnhum.2022.854175 (PMC9067321; doi:10.3389/fnhum.2022.854175)
Supplement: Supplementary file 2 [file Table_2.docx]

| Table 2  Participants Characteristics in the ITT sample at Baseline | | | | | | |
| --- | --- | --- | --- | --- | --- | --- |
|  | Total  Mean (SD) | AE  Mean (SD) | CCT  Mean (SD) | COMB  Mean (SD) | Control  Mean (SD) | Comparison Group  Mean (SD) |
| n total / n females | 92 / 58 | 30 / 16 | 24 / 17 | 23 / 17 | 15 / 8 | *Χ^2^*(3) = 3.61,  *p* = .306 |
| Age (years) | 57.91 (5.50) | 57.90 (5.22) | 57.63 (5.38) | 59.09 (5.79) | 56.60 (5.97) | H(3) = 1.66,  *p* = .645 |
| Years of education (years) | 12.76 (5.40) | 13.15 (5.56) | 12.04 (4.83) | 12.43 (5.04) | 13.60 (6.72) | H(3) = 1.05,  *p* = .788 |
| Vocabulary subtest (direct score-WAIS-II) | 44.04 (8.02) | 43.59 (8.91) | 43.88 (7.26) | 44.96 (7.40) | 43.80 (8.98) | F(3,87) = 0.14, *p* = .939 |
| AE = Aerobic Exercise group; CCT = Computerized Cognitive Training group; COMB = Combined group; WAIS-III = Wechsler Adult Intelligence Scale; X^2^ = chi square; H= Kruskall Wallis H test; F= Anova test | | | | | | |
