# Supplementary material for: Molecular and Brain Volume Changes Following Aerobic Exercise, Cognitive and Combined Training in Physically Inactive Healthy Late-Middle-Aged Adults: The Projecte Moviment Randomized Controlled Trial
Source: Front Hum Neurosci. 2022 Apr 20;16:854175. doi: 10.3389/fnhum.2022.854175 (PMC9067321; doi:10.3389/fnhum.2022.854175)
Supplement: Supplementary file 3 [file Table_3.docx]

| **Table 3**  Comparison of demographic data between ITT and PP sample | | | | | | | | | |
| --- | --- | --- | --- | --- | --- | --- | --- | --- | --- |
| GROUPS | | n total  /n females | | Age (years) | | Years of education | | Vocabulary subtest  WAIS-III | |
| Total Sample | ITT | 92 / 58 | *Χ^2^*(1) = 0.01,  *p* = .908 | 57.91 (5.50) | U = 3582.50, *p* = .567 | 12.76 (5.40) | U = 3637.50, *p* = .648 | 44.04 (8.02) | U = 3641.00, *p* = .891 |
|  | PP | 82 / 51 |  | 58.38 (5.47) |  | 12.52 (5.57) |  | 44.14 (8.30) |  |
| AE | ITT | 30 / 16 | *Χ^2^*(1) = 0.01,  *p* = .921 | 57.90 (5.22) | t(53) = -0.36, *p* = .723 | 13.15 (5.56) | t(53) = 0.46, *p* = .644 | 43.59 (8.91) | t(51) = -0.13, *p* = .897 |
|  | PP | 25 / 13 |  | 58.40 (5.12) |  | 12.44 (5.75) |  | 43.92 (9.53) |  |
| CCT | ITT | 24 / 17 | *Χ^2^*(1) = 0.01,  *p* = .924 | 57.63 (5.38) | t(45) = -0.19, *p* = .854 | 12.04 (4.83) | t(45) = -0.00, *p* = .999 | 43.88 (7.26) | t(45) = -0.18, *p* = .855 |
|  | PP | 23 / 16 |  | 57.91 (5.31) |  | 12.04 (4.94) |  | 44.26 (7.16) |  |
| COMB | ITT | 23 / 17 | *Χ^2^*(1) = 0.00,  *p* = .987 | 59.09 (5.79) | t(40) = -0.70, *p* = .489 | 12.43 (5.04) | U = 213.00, *p* = .888 | 44.96 (7.40) | t(40) = 0.18, *p* = .858 |
|  | PP | 19 / 14 |  | 60.32 (5.54) |  | 12.37 (5.43) |  | 44.53 (8.02) |  |
| AE = Aerobic exercise group; CCT = Computerized Cognitive Training group; COMB = Combined group; Control group is the same in ITT and PP sample; Mean (SD); X^2^ = chi square test, U = Mann Whitney U test; t = Student’s T-Test | | | | | | | | | |
