# Supplementary material for: Molecular and Brain Volume Changes Following Aerobic Exercise, Cognitive and Combined Training in Physically Inactive Healthy Late-Middle-Aged Adults: The Projecte Moviment Randomized Controlled Trial
Source: Front Hum Neurosci. 2022 Apr 20;16:854175. doi: 10.3389/fnhum.2022.854175 (PMC9067321; doi:10.3389/fnhum.2022.854175)
Supplement: Supplementary file 4 [file Table_4.docx]

| **Table 4.1**  Group comparison at Baseline: molecular markers at Level 1 | | | | | |
| --- | --- | --- | --- | --- | --- |
| Variables | Groups | n | Mean | SD | ANOVA / H de Kruskall Wallis |
| BDNF | AE | 20 | 4102.85 | 2875.20 | H(3) = 1.88, *p* = .597 |
|  | CCT | 23 | 5362.52 | 5648.96 |  |
|  | COMB | 17 | 3878.65 | 3521.72 |  |
|  | Control | 14 | 7036.71 | 6843.76 |  |
| TNF-α | AE | 25 | 0.76 | 0.22 | H(3) = 2.751, *p* = .432 |
|  | CCT | 23 | 0.76 | 0.23 |  |
|  | COMB | 19 | 0.77 | 0.21 |  |
|  | Control | 15 | 0.86 | 0.23 |  |
| HGF | AE | 25 | 1044.17 | 211.37 | H(3) = 1.785, *p* = .618 |
|  | CCT | 23 | 1088.67 | 294.30 |  |
|  | COMB | 19 | 1012.87 | 190.02 |  |
|  | Control | 15 | 1202.16 | 461.63 |  |
| ICAM | AE | 25 | 183.65 | 46.18 | H(3) = 3.533, *p* = .317 |
|  | CCT | 23 | 199.26 | 59.23 |  |
|  | COMB | 19 | 192.52 | 33.51 |  |
|  | Control | 15 | 209.43 | 46.50 |  |
| SDF-1a | AE | 25 | 1690.36 | 352.13 | H(3) = 8.809, *p* = .032 |
|  | CCT | 23 | 1573.73 | 357.46 |  |
|  | COMB | 19 | 1799.50 | 249.63 |  |
|  | Control | 15 | 1528.19 | 378.00 |  |
| AE = Aerobic exercise group; CCT = Computerized Cognitive Training group; COMB = Combined group. | | | | | |

| **Table 4.2**  Group comparison at Baseline: molecular markers at Level 2 | | | | | |
| --- | --- | --- | --- | --- | --- |
| Variables | Groups | n | Mean | SD | ANOVA / H de Kruskall Wallis |
| Ventricles | AE | 23 | 25685.17 | 7956.12 | H(3) = 1.79, *p* = .618 |
|  | CCT | 19 | 24435.68 | 8863.03 |  |
|  | COMB | 18 | 31135.22 | 18069.32 |  |
|  | Control | 14 | 32700.07 | 25718.23 |  |
| Total White Matter | AE | 23 | 166776.35 | 15774.44 | H(3) = 1.22, *p* = .747 |
|  | CCT | 19 | 164346.89 | 22250.41 |  |
|  | COMB | 18 | 168286.06 | 15338.20 |  |
|  | Control | 14 | 168022.29 | 17676.56 |  |
| Frontal Lobe | AE | 23 | 259245.04 | 26322.05 | F(3,70) = 1.06, *p* = .370 |
|  | CCT | 19 | 250909.84 | 29807.35 |  |
|  | COMB | 18 | 259359.61 | 24133.85 |  |
|  | Control | 14 | 267289.79 | 2341.,23 |  |
| Dorsolateral Prefrontal Cortex | AE | 23 | 28906.61 | 3102.48 | F(3,70) = 1.87, *p* = .144 |
|  | CCT | 19 | 28273.47 | 3796.36 |  |
|  | COMB | 18 | 29472.83 | 2679.67 |  |
|  | Control | 14 | 30755.14 | 2404.55 |  |
| Cingulate Cortex | AE | 23 | 37448.04 | 4553.70 | H(3) = 3.38, *p* = .336 |
|  | CCT | 19 | 36007.37 | 2865.67 |  |
|  | COMB | 18 | 37538.17 | 4944.15 |  |
|  | Control | 14 | 38820.86 | 4972-64 |  |
| Parietal Lobe | AE | 23 | 146732.48 | 13667.88 | H(3) = 0.95, *p* =.814 |
|  | CCT | 19 | 143949.68 | 13134.27 |  |
|  | COMB | 18 | 146130.44 | 13771.51 |  |
|  | Control | 14 | 147455.57 | 12404.42 |  |
| Precuneus | AE | 23 | 20884.17 | 2408.45 | H(3) = 3.18, *p* = .364 |
|  | CCT | 19 | 19815.32 | 2633.22 |  |
|  | COMB | 18 | 20679.00 | 2208.34 |  |
|  | Control | 14 | 20683.64 | 2889.26 |  |
| Temporal Lobe | AE | 23 | 170217.52 | 17892.31 | F(3,70) = 0.72, *p* = .545 |
|  | CCT | 19 | 165355.53 | 17157.27 |  |
|  | COMB | 18 | 168624.50 | 16474.14 |  |
|  | Control | 14 | 174026.07 | 16926.58 |  |
| Hippocampus | AE | 23 | 7349.96 | 899.55 | F(3,70) = 1.21, *p* = .313 |
|  | CCT | 19 | 7004.68 | 841.45 |  |
|  | COMB | 18 | 6947.56 | 704.79 |  |
|  | Control | 14 | 7280.57 | 641.06 |  |
| AE = Aerobic exercise group; CCT = Computerized Cognitive Training group; COMB = Combined group. | | | | | |

| **Table 4.3**  Group Comparison at Baseline: PA and CRF | | | | | |
| --- | --- | --- | --- | --- | --- |
| Variables | Groups | N | Mean | SD | ANOVA / H de Kruskall Wallis |
| CRF | AE | 19 | 25.25 | 10.16 | F(3,67) = 1.08, *p* = .362 |
|  | CCT | 20 | 26.11 | 12.50 |  |
|  | COMB | 17 | 27.34 | 8.75 |  |
|  | Control | 15 | 20.65 | 12.69 |  |
| S-PA | AE | 25 | 451.98 | 699.40 | H(3) = 2.92, *p* = .404 |
|  | CCT | 23 | 439.83 | 713.63 |  |
|  | COMB | 19 | 778.79 | 908.77 |  |
|  | Control | 15 | 366.80 | 618.17 |  |
| NS-PA | AE | 25 | 5595.73 | 3918.34 | H(3) = 7.96, *p* = .047 |
|  | CCT | 23 | 9113.74 | 7104.64 |  |
|  | COMB | 19 | 10295.68 | 6159.04 |  |
|  | Control | 15 | 7038.40 | 6628.45 |  |
| AE = Aerobic exercise group; CCT = Computerized Cognitive Training group; COMB = Combined group; S-PA = Sportive Physical Activity; NS-PA = Non Sportive Physical Activity; Total-PA = Total Physical Activity; CRF = Cardiorespiratory Fitness. | | | | | |

| **Table 4.4**  Group comparison at Baseline: z-scores of Cognitive Domains | | | | | |
| --- | --- | --- | --- | --- | --- |
| Variables | Groups | n | Mean | SD | ANOVA / H de Kruskall Wallis |
|  |  |  |  |  |  |
| Executive Function | AE | 24 | -0.01 | 0.72 | F(3,75) = 0.81, *p* = .492 |
|  | CCT | 23 | 0.16 | 0.60 |  |
|  | COMB | 19 | -0.08 | 0.50 |  |
|  | Control | 13 | -0.15 | 0.81 |  |
| Flexibility | AE | 25 | 0.10 | 1.05 | H(3) = 1.53, *p* = .676 |
|  | CCT | 23 | -0.10 | 1.06 |  |
|  | COMB | 19 | -0.03 | 0.80 |  |
|  | Control | 14 | 0.04 | 1.12 |  |
| Fluency | AE | 25 | 0.02 | 0.91 | F(3,77) = 1.19, *p* = .318 |
|  | CCT | 23 | 0.22 | 0.76 |  |
|  | COMB | 19 | -0.10 | 0.82 |  |
|  | Control | 14 | -0.28 | 0.80 |  |
| Inhibition | AE | 24 | -0.06 | 1.03 | F(3,77) = 1.18, *p* = .323 |
|  | CCT | 23 | 0.32 | 0.86 |  |
|  | COMB | 19 | -0.16 | 1.18 |  |
|  | Control | 15 | -0.20 | 0.89 |  |
| Working Memory | AE | 25 | -0.06 | 1.10 | H(3) = 1.29, *p* = .732 |
|  | CCT | 23 | 0.14 | 1.05 |  |
|  | COMB | 19 | -0.01 | 0.69 |  |
|  | Control | 15 | -0.11 | 1.16 |  |
| Visuospatial Function | AE | 25 | 0.14 | 0.93 | H(3) = 3.40, *p* = .334 |
|  | CCT | 23 | -0.20 | 1.04 |  |
|  | COMB | 19 | -0.19 | 1.24 |  |
|  | Control | 15 | 0.32 | 0.62 |  |
| Language | AE | 25 | 0.01 | 1.09 | H(3) = 0.71, *p* = .870 |
|  | CCT | 23 | -0.04 | 1.08 |  |
|  | COMB | 19 | -0.10 | 0.91 |  |
|  | Control | 15 | 0.16 | 0.89 |  |
| Attention-Speed | AE | 24 | -0.02 | 1.01 | H(3) = 2.34, *p* = .506 |
|  | CCT | 23 | 0.13 | 0.65 |  |
|  | COMB | 19 | -0.17 | 0.68 |  |
|  | Control | 14 | 0.09 | 0.44 |  |
| Attention | AE | 25 | 0.03 | 0.95 | F(3,77) = 0.70, *p* = .553 |
|  | CCT | 23 | 0.15 | 0.71 |  |
|  | COMB | 19 | -0.20 | 0.76 |  |
|  | Control | 14 | 0.03 | 0.62 |  |
| Speed | AE | 24 | -0.12 | 1.21 | H(3) = 1.64, *p* = .650 |
|  | CCT | 23 | 0.11 | 0.77 |  |
|  | COMB | 19 | -0.12 | 0.74 |  |
|  | Control | 15 | 0.17 | 0.32 |  |
| Memory | AE | 25 | 0.03 | 0.70 | H(3) = 0.95, *p* = .815 |
|  | CCT | 23 | 0.13 | 0.66 |  |
|  | COMB | 19 | -0.14 | 0.92 |  |
|  | Control | 14 | -0.09 | 0.95 |  |
| Visual Memory | AE | 25 | 0.17 | 1.05 | F(3,78) = 1.00, *p* =.398 |
|  | CCT | 23 | 0.06 | 0.98 |  |
|  | COMB | 19 | -0.34 | 1.17 |  |
|  | Control | 15 | 0.04 | 0.65 |  |
| Verbal Memory | AE | 25 | -0.04 | 0.71 | H(3) = 0.70, *p* = .874 |
|  | CCT | 23 | 0.16 | 0.79 |  |
|  | COMB | 19 | -0.04 | 1.08 |  |
|  | Control | 14 | -0.14 | 1.29 |  |
| Global Cognitive Function | AE | 23 | 0.01 | 0.73 | F(3,74) = 0.51, *p* = .675 |
|  | CCT | 23 | .011 | 0.56 |  |
|  | COMB | 19 | -0.13 | 0.57 |  |
|  | Control | 13 | -0.03 | 0.59 |  |
| AE = Aerobic exercise group; CCT = Computerized Cognitive Training group; COMB = Combined group. | | | | | |
